# Performance of TcI/TcVI/TcII Chagas-Flow ATE-IgG2a for universal and genotype-specific serodiagnosis of *Trypanosoma cruzi* infection

**DOI:** 10.1371/journal.pntd.0005444

**Published:** 2017-03-23

**Authors:** Glaucia Diniz Alessio, Fernanda Fortes de Araújo, Denise Fonseca Côrtes, Policarpo Ademar Sales Júnior, Daniela Cristina Lima, Matheus de Souza Gomes, Laurence Rodrigues do Amaral, Marcelo Antônio Pascoal Xavier, Andréa Teixeira-Carvalho, Olindo Assis Martins-Filho, Marta de Lana

**Affiliations:** 1 Laboratório de Doença de Chagas, Núcleo de Pesquisas em Ciências Biológicas (NUPEB), Instituto de Ciências Exatas e Biológicas (ICEB), Universidade Federal de Ouro Preto (UFOP), Ouro Preto, Minas Gerais, Brazil; 2 Grupo Integrado de Pesquisas em Biomarcadores, Centro de Pesquisas René Rachou (CPqRR-FIOCRUZ/ MG), Belo Horizonte, Minas Gerais, Brazil; 3 Grupo de Genômica Funcional e Proteômica de *Leishmania spp* e *Trypanosoma cruzi*, Centro de Pesquisas René Rachou (CPqRR-FIOCRUZ/ MG), Belo Horizonte, Minas Gerais, Brazil; 4 Laboratório de Bioinformática e Análises Moleculares, Universidade Federal de Uberlândia, INGEB/FACOM, Campus Patos de Minas, Patos de Minas, Minas Gerais, Brazil; 5 Grupo de Pesquisas Clínicas e Políticas Públicas em Doenças Infecciosas e Parasitárias, Centro de Pesquisas René Rachou (CPqRR-FIOCRUZ/ MG), Belo Horizonte, Minas Gerais, Brazil; Instituto de Investigaciones Biotecnológicas, ARGENTINA

## Abstract

Distinct *Trypanosoma cruzi* genotypes have been considered relevant for patient management and therapeutic response of Chagas disease. However, typing strategies for genotype-specific serodiagnosis of Chagas disease are still unavailable and requires standardization for practical application. In this study, an innovative TcI/TcVI/TcII Chagas Flow ATE-IgG2a technique was developed with applicability for universal and genotype-specific diagnosis of *T*. *cruzi* infection. For this purpose, the reactivity of serum samples (percentage of positive fluorescent parasites-PPFP) obtained from mice chronically infected with TcI/Colombiana, TcVI/CL or TcII/Y strain as well as non-infected controls were determined using amastigote-AMA, trypomastigote-TRYPO and epimastigote-EPI in parallel batches of TcI, TcVI and TcII target antigens. Data demonstrated that “α-TcII-TRYPO/1:500, cut-off/PPFP = 20%” presented an excellent performance for universal diagnosis of *T*. *cruzi* infection (AUC = 1.0, Se and Sp = 100%). The combined set of attributes “α-TcI-TRYPO/1:4,000, cut-off/PPFP = 50%”, “α-TcII-AMA/1:1,000, cut-off/PPFP = 40%” and “α-TcVI-EPI/1:1,000, cut-off/PPFP = 45%” showed good performance to segregate infections with TcI/Colombiana, TcVI/CL or TcII/Y strain. Overall, hosts infected with TcI/Colombiana and TcII/Y strains displayed opposite patterns of reactivity with “α-TcI TRYPO” and “α-TcII AMA”. Hosts infected with TcVI/CL strain showed a typical interweaved distribution pattern. The method presented a good performance for genotype-specific diagnosis, with global accuracy of 69% when the population/prototype scenario include TcI, TcVI and TcII infections and 94% when comprise only TcI and TcII infections. This study also proposes a receiver operating reactivity panel, providing a feasible tool to classify serum samples from hosts infected with distinct *T*. *cruzi* genotypes, supporting the potential of this method for universal and genotype-specific diagnosis of *T*. *cruzi* infection.

## Author summary

Chagas disease remains a significant public health issue infecting 6–7 million people worldwide. The factors influencing the clinical heterogeneity of Chagas disease have not been elucidated, although it has been suggested that different clinical outcome may be associated with the genetic diversity of *T*. *cruzi* isolates. Moreover, differences in therapeutic response of distinct *T*. *cruzi* genotypes have been also reported. Typing strategies for genotype-specific diagnosis of Chagas disease to identify the *T*. *cruzi* discrete typing units (DTU) have already been developed, including biochemical and molecular methods, however the techniques have limitations. The majority of these methods can not directly be performed in biological and clinical samples. In addition, it has been proposed that parasite isolates from blood may not necessarily represent the full set of strains current in the individual as some strains can be confined to tissues. The improvement of genotype-specific serology to identify the *T*. *cruzi* DTU(s) present in a given host may provide a useful tool for clinical studies. In the present investigation, we developed an innovative TcI/TcVI/TcII Chagas Flow ATE-IgG2a technique with applicability for universal and genotype-specific diagnosis of *T*. *cruzi* infection that may contribute to add future insights for genotype-specific diagnosis of Chagas disease.

## Introduction

*Trypanosoma cruzi*, the etiological agent of Chagas disease [1] infects 6–7 million people worldwide, mainly in Latin America causing serious consequences for public health and national economies [2]. Geographical variations in the prevalence of clinical forms and morbidity of Chagas disease in different countries have been recorded [3]. Although the factors underlying the clinical heterogeneity of Chagas disease are still not completely understood, it has been suggested that different clinical outcome may be associated with the genetic diversity of *T*. *cruzi* isolates observed in the Americas [4]. Moreover, differences in therapeutic response of distinct *T*. *cruzi* genotypes have been also reported previously in mice infection [5–8].

Typing strategies for genotype-specific diagnosis of Chagas disease to identify the six *T*. *cruzi* discrete typing units (DTU), named TcI, TcII, TcIII, TcIV, TcV and TcVI [9] have already been developed, including biochemical and molecular methods [4]. However, none of these methods allows a full resolution when used individually and a combinatory three-marker sequential typing strategy is usually required to confirm the *T*. *cruzi* genotype [10–12]. Straightforward, genotyping methods to identify the *T*. *cruzi* DTUs are currently available, but research is still required to optimize sensitivity and simplify methods so that they can be easily applied in clinical laboratories. In fact, molecular methods require a measurable parasite load to directly identify *T*. *cruzi* DTUs in samples. Because of this, the approaches used for *T*. *cruzi* genotyping requires parasite isolation by hemoculture/xenoculture followed by in vitro growth that may lead to clonal selection [13–16].

A feasible solution to overcome these problems is the design and development of genotype-specific serology to provide a current/historical profile of *T*. *cruzi* DTUs infecting an individual patient [17–20]. Moreover, genotypic-specific serodiagnosis has the potential to predict therapeutic response and provide insights upon re-activation episodes.

Recently, a flow cytometry-based assay, named Chagas-Flow ATE (Amastigote, Trypomastigote and Epimastigote), has been developed for simultaneous measurement of anti-amastigote, anti-trypomastigote and anti-epimastigote antibodies displaying high performance for the diagnosis and post-therapeutic monitoring of Chagas disease [21]. Aiming at optimizing the Chagas-Flow ATE for universal and genotypic-specific diagnosis of *T*. *cruzi* infection, the present study proposed the development of modified Chagas-Flow ATE, using parallel batches of distinct *T*. *cruzi* genotypes as target antigens. Standard *T*. *cruzi* strains, representative of three major genotypes (TcI, TcII and TcVI) were used to setup the Chagas-Flow ATE-IgG2a methodology.

High-dimensional data handling were applied to select the sets attributes (“target-antigen/serum dilution/cut-off”) applicable for universal and genotypic-specific diagnosis of *T*. *cruzi*-infection. A receiver operating reactivity panel was proposed as a feasible tool to identify hosts infected with distinct *T*. *cruzi* genotypes. The results demonstrated the high-quality performance of TcI/TcVI/TcII Chagas-Flow ATE-IgG2a for universal and genotype-specific diagnosis of *T*. *cruzi* infection.

## Methods

### Ethics statement

All animals included in this study were maintained at the Animal Science Center of the Universidade Federal de Ouro Preto, Ouro Preto, MG, Brazil, in strict accordance with the Brazilian College of Animal Experimentation Guidelines for ethical conduct in use of animals in research. Efforts were performed to reduce animal suffering. The study protocols were approved by the Ethics Committee on Animal Experimentation of the Federal University of Ouro Preto (Protocol approval numbers #2013/48 from December, 6^th^, 2013 for the experimental infection and collected blood by ocular plexus puncture in mice).

### *Trypanosoma cruzi* strains

Standard *T*. *cruzi* strains, representative of three major genotypes [9], involved in the domestic cycle of Chagas disease in Brazil, were used to setup the TcI/TcVI/TcII Chagas-Flow ATE-IgG2a methodology for the serodiagnosis of *T*. *cruzi* infection. The Colombiana, acronyms “COL” (TcI) [22], CL (TcVI) [23] and Y (TcII) *T*. *cruzi* strains were used in this study [24]. All isolates were obtained from the *T*. *cruzi* cryobank at Grupo de Genômica Funcional e Proteômica de *Leishmania spp* e *Trypanosoma cruzi*, Centro de Pesquisas René Rachou (CPqRR-FIOCRUZ/ MG). The *T*. *cruzi* strains were maintained by consecutive *in vivo* passages in Swiss female mice. Blood samples obtained from infected mice were used for experimental infection as well as for preparation of target antigens (amastigote-AMA, trypomastigote-TRYPO and epimastigote-EPI) used on each TcI/TcVI/TcII Chagas-Flow ATE-IgG2a platform.

### Experimental infection with TcI, TcVI and TcII *T*. *cruzi* genotypes

Female Swiss mice (n = 118, 28–30 days old), obtained from the Animal Science Centre at the Universidade Federal de Ouro Preto (UFOP), MG, Brazil, were maintained in temperature-controlled room with access to water and food *ad libitum*. Animals were subdivided into four groups referred as *T*. *cruzi*-infected TcI/Colombiana/COL strain (n = 36), TcVI/CL strain (n = 36) or TcII/Y strain (n = 36) as well as non-infected mice (NI, n = 10).

The infection was confirmed in all *T*. *cruzi*-infected mice, by positivity at fresh blood examination performed at day 7, 10 or 15 post-infection. The serum samples used for the TcI/TcVI/TcII Chagas-Flow ATE-IgG2a serology were prepared from whole blood samples collected by ocular plexus puncture. Samples were collected from non-infected controls and *T*. *cruzi*-infected mice (day 90 and day180 post-infection) were inactivated at 56°C for 30 min and stored at -20°C until use. Considering the animal mortality during the experimental timeline, the final number of animals/group were (TcI/Colombiana strain, n = 29, TcVI/CL strain, n = 29, TcII/Y strain, n = 35 and NI, n = 10).

### Preparation of AMA, TRYPO, EPI (ATE) target antigens from TcI, TcVI and TcII *T*. *cruzi* genotypes

**Fig 1:**
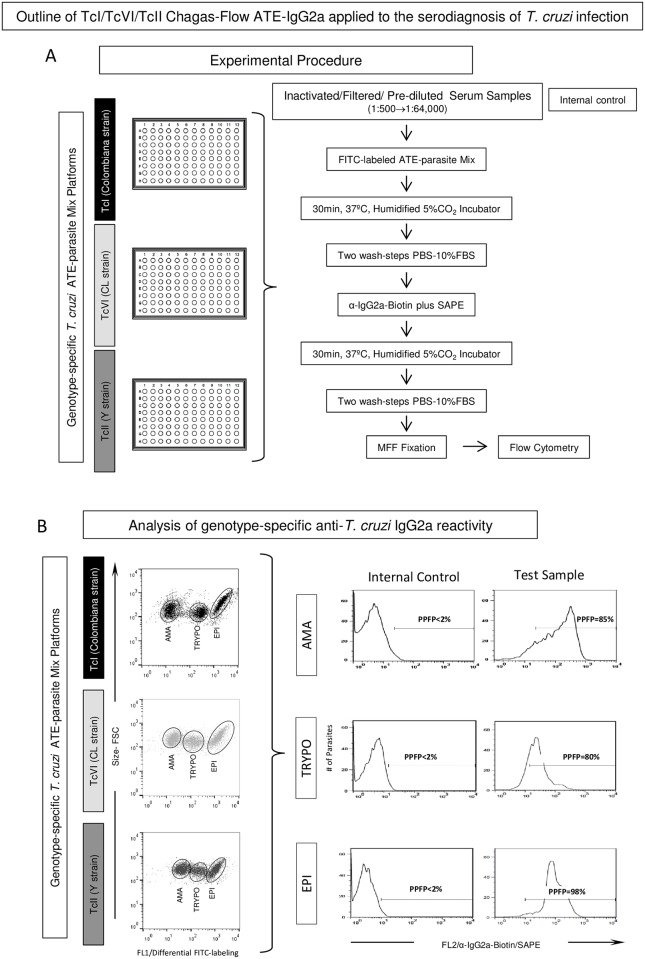
Outline of TcI/TcVI/TcII Chagas-Flow ATE-IgG2a for serodiagnosis of *Trypanosoma cruzi* infection. (A) The experimental procedure display a schematic representation of genotype-specific *T*. *cruzi* ATE-parasite Mix Platforms using TcI (Colombiana strain) = black bar, TcVI (CL strain) = light gray bar and TcII (Y strain) = dark gray bar antigens in separate batches. (B) Representative gating strategies used to select the target antigens (amastigote-AMA, trypomastigote-TRYPO and epimastigote-EPI) on each Chagas-Flow ATE-IgG2a platform and the histograms employed to quantify the genotype-specific anti-*T*. *cruzi* IgG2a reactivity, expressed by the percentage of positive fluorescent parasites (PPFP), based on the positivity limit (PPFP<2%), set based on the internal control.

The amastigote/trypomastigote/epimastigote forms of TcI, TcVI and TcII *T*. *cruzi* genotypes were obtained as described previously by Alessio *et al*. (2014) [21]. Enriched trypomastigotes (TRYPO) and amastigote (AMA) preparations were obtained from desynchronized *in vitro* tissue cultures (L929 cell-line) harvested at day 4–6 and 8–15 post-inoculation, respectively. Epimastigote forms were obtained at log-phase growth of axenic culture in liver infusion tryptose medium [25]. Live amastigotes and trypomastigotes as well as fixed epimastigotes forms were stained with fluorescein isothiocyanate (FITC) as described by Alessio *et al*. (2014) [21]. Briefly, AMA/TRYPO mix and EPI suspensions (1x10^7^ parasites/mL) were stained with FITC (100μg/mL for TcI/Colombiana strain and 200μg/mL for TcVI/CL strain and TcII/Y strain) for 30 min at 37°C. After staining, AMA/TRYPO mix were kept at 37°C for 60min and EPI preparation stored at 4°C for 24h prior to use. The three FITC-labeled parasite preparations were mixed accordingly to obtain an equivalent proportion of AMA (33%), TRYPO (33%) and EPI (33%) in the final ATE-parasite Mix Platforms, monitored by flow cytometry checking performed prior use. The FITC-labeling approach led to a differential staining phenomenon previously described by Alessio *et al*., (2014) [21] that allowed the segregation of AMA, TRYPO and EPI organisms in distinct clusters, based on the FITC (Fluorescence 1- FL1) *vs* Forward Scatter (FSC) dot plot distribution (Fig 1).

### TcI/TcVI/TcII Chagas-Flow ATE-IgG2a serodiagnosis of *T*. *cruzi* infection

An outline of the TcI/TcVI/TcII Chagas-Flow ATE-IgG2a applied to the serodiagnosis of *T*. *cruzi* infection is provided in the Fig 1. The method comprises two steps referred as: i) Experimental Procedure (Fig 1A) and ii) Analysis of genotype-specific anti-*T*. *cruzi* IgG2a reactivity (Fig 1B).

#### Experimental procedure

The TcI/TcVI/TcII Chagas-Flow ATE-IgG2a serodiagnosis was performed as described previously by Alessio *et al*. (2014) [21] modified as follows: frozen serum samples were thawed from -20°C storage, filtered through 0.22μm syringe filter and submitted to serial dilution (1:500 to 1:64,000) with phosphate-buffered-saline supplemented with 10% fetal bovine serum (PBS-10%FBS) in a U-bottom 96-well plate. A final volume of 50μL of pre-diluted serum samples were incubated with 50μL of each ATE-parasite Mix preparation (TcI, TcVI and TcII genotype-specific platforms, in parallel batches) for 30 min at 37°C in a 5% CO_2_ humidified incubator. Following incubation, parasites were washed twice with PBS-10%FBS and the supernatant discarded. The pellet of parasite mix was re-suspended and incubated with 50μL biotin-conjugated anti-mouse IgG2a that is equivalent of human IgG1 (1:3,000 in PBS-10%FBS) plus 20μL of secondary reagents (phycoerytrin-conjugated streptavidin-SAPE, 1:800 in PBS-10%FBS) for 30 min at 37°C in a 5% CO_2_ humidified incubator. Parasites were washed once with PBS-10%FBS and fixed with 200μL of FACS fixing solution (10g/L of paraformaldehyde, 10.2g/L of sodium cacodylate and 6.65g/L of sodium chloride, pH 7.2), and store at 4°C until flow cytometric data acquisition in a FACSCan flow cytometer (Beckton Dickinson, USA). An internal control (“second step reagents control” = anti-mouse IgG2a-biotin+SAPE) to monitor unspecific bindings was included in all experimental batches, in which the ATE-parasite Mix preparations were incubated in the absence of mouse serum but in the presence of secondary reagents. A total of 10,000 events were acquired for each tested serum dilution. Acquisition was performed with appropriate instrument settings on log scale (FSC = E00, Side Scatter -SSC = 427, threshold = 400; FL1 = 620 and FL2 = 500). Data were stored for off-line analysis.

#### Analysis of genotype-specific anti-*T*. *cruzi* IgG2a reactivity

The FlowJo software version 10.1 (TreeStar, San Diego, CA, USA) was used for off-line data analysis. The genotype-specific reactivity of anti-*T*. *cruzi* IgG2a was performed for each ATE-parasite mix platform—TcI (Colombiana strain), TcVI (CL strain) and TcII (Y strain). Appropriate gating strategies were used to select the target antigens (amastigote-AMA, trypomastigote-TRYPO and epimastigote-EPI) on each Chagas-Flow ATE-IgG2a platform, based on the differential FITC-labeling features of AMA, TRYPO and EPI. Following the selection of target-antigens, one-dimensional histograms were employed to quantify the genotype-specific anti-*T*. *cruzi* IgG2a reactivity, based on the positivity limit (PPFP<2%), set based on the internal control. The results were expressed as percentage of positive fluorescent parasites (PPFP) for each tested sample dilution (Fig 1B).

### Data mining and analysis

Data mining for universal and genotype-specific diagnosis of *T*. *cruzi* infection was first performed by non-parametric Kruskal—Wallis test followed by Dunns' multiple comparison post-test to compare the overall reactivity profile of TcI/TcVI/TcII Chagas-Flow ATE-IgG2a. Significant differences were considered at p ≤ 0.05. The performance indices (global accuracy defined by the area under the curve-AUC, sensitivity-Se and specificity-Sp) for the pair of attributes (“target antigen/serum dilution”) selected for universal diagnosis purposes were determined by the receiver operating characteristic (ROC) curve, scatter plot distribution and Two-Graph ROC curve (TG-ROC) analysis. Histogram plot distributions and nonlinear regression analysis was used for comparative analysis of pair of attributes (“target antigen/serum dilution”) selected for genotypic-specific diagnosis purposes. The global median was calculated for each pair of attributes (“target antigen/serum dilution”) to define putative cut-off edges to segregate the reactivity amongst *T*. *cruzi*-infected hosts. Scatter plot distribution was used for performance analysis of sets of selected attributes (“target-antigen/serum dilution/cut-off”) applicable for genotypic-specific diagnosis of *T*. *cruzi*-infection. The GraphPad Prism software, Version 5.0 (San Diego, CA, USA) was used for statistical analysis and graphic arts.

Decision trees were built for the set of selected attributes (“target-antigen/serum dilution/cut-off”) to create algorithms (root and branch attributes) to classify *T*. *cruzi* in distinct population/prototype scenarios (TcI-infection/Colombiana *vs* TcVI/CL *vs* TcII/Y) and (TcI-infection/Colombiana *vs* TcII/Y). The WEKA software (Waikato Environment for Knowledge Analysis, version 3.6.11, University of Waikato, New Zealand) was used for decision tree construction.

Step-wise discriminant analysis was applied to determine the global accuracy and the leave-one-out-cross-validation-LOOCV values. The R-project for statistical computing software, Version 3.0.1 was used for discriminant analysis. The algorithm C4.5 was used to build the decision tree using an implementation named J48. This method analyzed all characteristics to select a minimum set of markers that could efficiently separate study groups.

## Results

### Overall reactivity profile of TcI/TcVI/TcII Chagas-Flow ATE-IgG2a for universal and genotypic-specific diagnosis of *T*. *cruzi* infection

**Fig 2:**
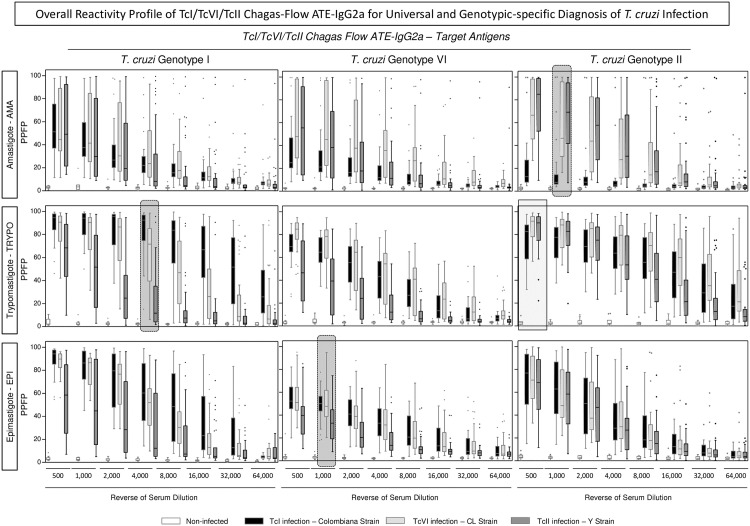
Overall reactivity profile of TcI/TcVI/TcII Chagas-Flow ATE-IgG2a for universal and genotypic-specific diagnosis of *T*. *cruzi* infection. The Chagas-Flow ATE-IgG2a reactivity was determined for sera samples from *T*. *cruzi*-infected mice, including TcI/TcVI/TcII genotype-representative strains, including: TcI/Colombiana strain (black dotted frame, n = 29), TcVI/CL strain (light gray dotted frame, n = 29) and TcII/Y strain (dark gray dotted frame, n = 35) as well as non-infected mice (white dotted frame, n = 10). Genotype-specific IgG2a reactivity to each target-antigen (amastigote-AMA, trypomastigote-TRYPO and epimastigote-EPI) from *T*. *cruzi* genotype I (left panels), genotype VI (middle panels) and genotype II (right panels) was assessed at eight serum dilutions (1:500 to 1:64,000). The results are expressed as the percentage of positive fluorescent parasites (PPFP), using the box plot format, stretching from min to max values with outliers represented by gray-shaded dots and the box defining the 25^th^ and 75^th^ percentile and the median value (line across the box). Comparative analyses were performed by the Kruskal-Wallis followed by Dunn’s post test for multi-group comparisons. Significant differences were considered at p<0.05. The light gray continuous rectangle selects the pair of attributes (“target antigen/serum dilution”) with the most consistent ability to discriminate non-infected mice from all *T*. *cruzi*-infected hosts (Colombiana, CL and Y strains). Therefore, these features (anti-TcII TRYPO reactivity at 1:500) were selected for universal diagnosis of *T*. *cruzi* infection. The dark gray dotted frame select the pair of attributes “target antigen/serum dilution” with the most promising perspective to distinguish the reactivity of sera samples amongst host infected with Colombiana, CL or Y *T*. *cruzi* strains. Therefore, these features (anti-TcII AMA reactivity at 1:1,000; anti-TcI TRYPO reactivity at 1:4,000 and anti-TcVI EPI reactivity at 1:1,000) were selected for genotype-specific diagnosis of *T*. *cruzi* infection.

The overall profiles of TcI/TcVI/TcII Chagas-Flow ATE-IgG2a reactivity observed for *T*. *cruzi* infected mice (TcI/Colombiana strain, TcVI/CL strain and TcII/Y strain) and non-infected controls are presented in the Fig 2. The reactivity of individual samples were assessed for distinct target-antigen (amastigote-AMA, trypomastigote-TRYPO and epimastigote-EPI) from *T*. *cruzi* genotype I—Colombiana strain (Fig 2—left panels), genotype VI—CL strain (Fig 2—middle panels) and genotype II—Y strain (Fig 2—right panels) along the titration curves (serum dilutions ranging from 1:500 to 1:64,000).

Comparative analysis allowed the selection of pair of attributes (“target antigen/serum dilution”) with the most promising perspective to be used for universal and genotypic-specific diagnosis of *T*. *cruzi* infection.

The pair of attributes “anti-TcII TRYPO reactivity at 1:500” presented the highest significant difference between non-infected mice and all *T*. *cruzi*-infected hosts (TcI/Colombiana, TcVI/CL and TcII/Y strains), and therefore was further evaluated for universal diagnosis purpose (Fig 2—light gray continuous rectangle).

The pairs of attributes with putative applicability to genotype-specific diagnosis of *T*. *cruzi* infection comprise: (“anti-TcII AMA reactivity at 1:1,000”; “anti-TcI TRYPO reactivity at 1:4,000” and “anti-TcVI EPI reactivity at 1:1,000”). The pair of attributes “anti-TcII AMA reactivity at 1:1,000” presented the highest ability to distinguish the lower reactivity of hosts infected with TcI/Colombiana strain from the higher reactivity observed for hosts infected with TcVI/CL or TcII/Y strains (Fig 2—right dark gray dotted frame). The pair of attributes “anti-TcI TRYPO reactivity at 1:4,000” presented the highest ability to discriminate lower reactivity of hosts infected with TcII/Y strain from the intermediate reactivity observed for hosts infected with TcVI/CL strain and the higher reactivity observed for hosts infected with TcI/Colombiana strain (Fig 2—left dark gray dotted frame). The pair of attributes “anti-TcVI EPI reactivity at 1:1,000” presented the most relevant potential to distinguish the lower reactivity of hosts infected with TcII/Y strain from the higher reactivity observed for hosts infected with TcI/Colombiana or TcVI/CL *T*. *cruzi* strains (Fig 2—middle dark gray dotted frame). Together, these pairs of attributes were selected for further performance assessment applicable to the genotype-specific diagnosis of *T*. *cruzi* infection.

### Performance of TcI/TcVI/TcII Chagas-Flow ATE-IgG2a for universal diagnosis of *T*. *cruzi* infection

**Fig 3:**
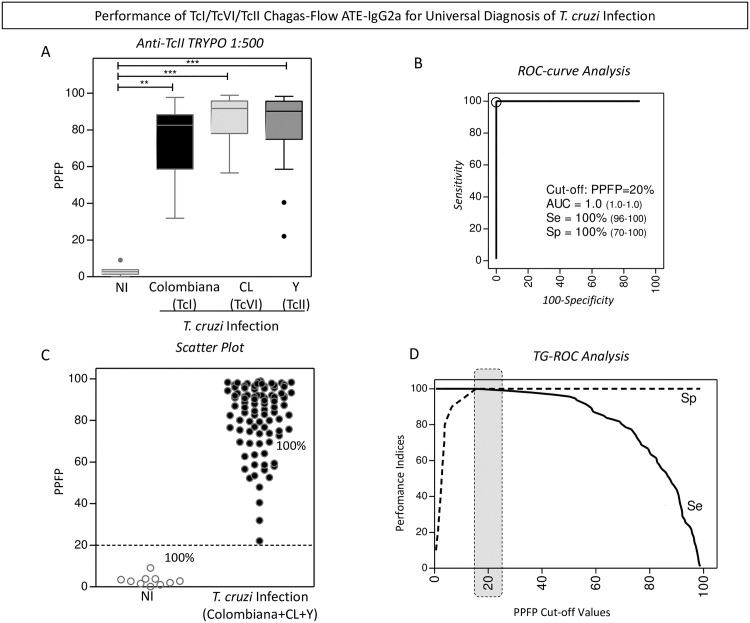
Performance of TcI/TcVI/TcII Chagas-Flow ATE-IgG2a for universal diagnosis of *T*. *cruzi* infection. (A) The anti-TcII TRYPO reactivity at 1:500, pre-selected as attributes pairs for universal *T*. *cruzi* infection diagnosis, were compared by Kruskal-Wallis followed by Dunn’s post test for multi-group comparisons and significant differences at *p <0.05, **p<0,001 and ***p<0,0001, highlighted by connecting lines. Data are expressed as median PPFP values for non-infected mice (white bar) and *T*. *cruzi*-infected hosts (TcI/Colombiana strain = black bar, TcVI/CL strain = light gray bar and TcII/Y strain = dark gray bar). The similarity amongst the anti-TcII TRYPO IgG2a reactivity at 1:500 observed for the three *T*. *cruzi* infected groups (Colombiana + CL + Y strains) allows the establishment of a single group referred to as *T*. *cruzi* infected hosts (n = 93) and the performance of the TcI/TcVI/TcII Chagas-Flow ATE-IgG2a in the universal diagnosis of *T*. *cruzi* infection carried out as compared to a group of non-infected mice (NI, n = 10). (B) ROC-curve analysis was applied to define the appropriated cut-off to discriminate the PPFP values from NI and *T*. *cruzi*-infected host (Colombiana + CL + Y strains). Additional performance indices were also calculated and provided in the figure, including the area under the curve (AUC), defined as global accuracy, the sensitivity (Se) and the specificity (Sp). (C) Representative scatter plot illustrates the ability of the selected set of attributes (“target-antigen/serum dilution/cut-off”) to discriminate the reactivity of the sera from non-infected (NI) and *T*. *cruzi*-infected hosts (Colombiana+CL+Y). The dotted line represented the cut-off of PPFP = 20% defined by the ROC-curve analysis. (D) TG-ROC analysis was also performed to confirm the cut-off selection at higher “Se” and “Sp”, highlighted by dark gray dotted frame.

The performance of the pre-selected pair of attributes “anti-TcII TRYPO reactivity at 1:500” applied to the universal diagnosis of *T*. *cruzi* infection is present in the Fig 3.

Comparative analysis demonstrated that the median value of “anti-TcII TRYPO reactivity at 1:500” differ significantly between non-infected mice and all *T*. *cruzi*-infected hosts (TcI/Colombiana, TcVI/CL and TcII/Y strains) (Fig 3A).

ROC curve analysis indicated the PPFP value of 20% as the cut-off edge with excellent performance indices (area under the curve-AUC = 1.0 along with Sensitivity-Se and Specificity-Sp of 100%) (Fig 3B). Scatter plot distribution of individual values illustrates the ability of this set of attributes to completely segregate the serum samples of the NI and *T*. *cruzi*-infected hosts (Fig 3C). Additional analysis by TG-ROC confirmed the selected PPFP value of 20% as the best cut-off for universal diagnosis of *T*. *cruzi* infection using the selected set of attributes (Fig 3D).

### Overall reactivity of TcI/TcVI/TcII Chagas-Flow ATE-IgG2a applied for genotype-specific diagnosis of *T*. *cruzi* infection

**Fig 4:**
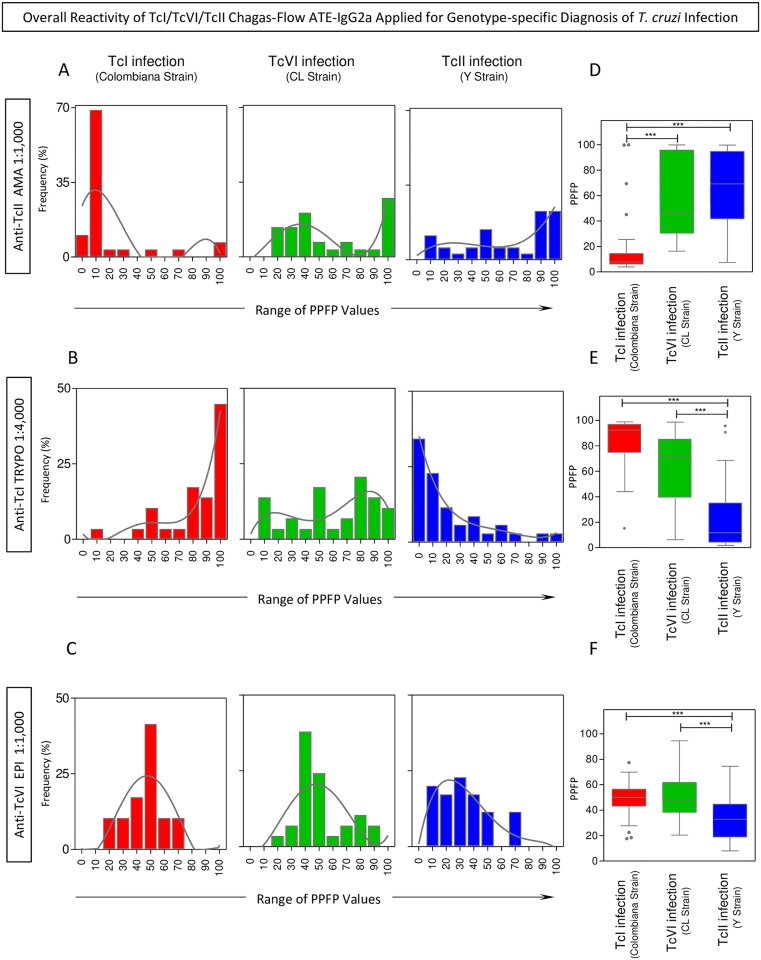
Overall reactivity of TcI/TcVI/TcII Chagas-Flow ATE-IgG2a applied for genotype-specific diagnosis of *T*. *cruzi*-infection. (A) The anti-TcII AMA reactivity at 1:1,000, (B) the anti-TcI TRYPO reactivity at 1:4,000 and (C) the anti-TcVI EPI reactivity at 1:1,000, pre-selected as attributes for genotypic-specific diagnosis of *T*. *cruzi*-infection, were further evaluated by histogram plot distributions and trendlines built by nonlinear regression. The results were expressed as the proportion of samples displaying a given PPFP values amongst *T*. *cruzi*-infected hosts, including: TcI/Colombiana strain (red bar, n = 29), TcVI/CL strain (green bar, n = 29) and TcII/Y strain (blue bar, n = 35). The reactivity amongst *T*. *cruzi*-infected groups was further compared for each pair of attributes, including: (D) anti-TcII AMA reactivity at 1:1,000, (E) the anti-TcI TRYPO reactivity at 1:4,000 and (F) the anti-TcVI EPI reactivity at 1:1,000. The results are expressed as median PPFP values in box plot format with the outliers underscored by gray-shaded dots. Data analyses were carried out by Kruskal-Wallis followed by Dunn’s post test for multi-group comparisons The significant differences were indicated by asterisk at *p <0.05, **p<0,001 and ***p<0,0001 and highlighted by connecting lines.

The overall reactivity profile of the pre-selected pairs of attributes (“anti-TcII AMA reactivity at 1:1,000”; “anti-TcI TRYPO reactivity at 1:4,000” and “anti-TcVI EPI reactivity at 1:1,000”) are shown in the Fig 4.

Data mining was carried out by histogram graph and trendlines drawn by non-linear regression analysis. The results showed that the “anti-TcII AMA reactivity at 1:1,000” of serum samples from hosts infected with TcI/Colombiana strain displayed a nearly unimodal distribution in the region of PPFP values = 10%, contrasting with the bimodal distribution of serum samples from hosts infected with TcVI/CL and TcII/Y strains that shows a shift towards higher PPFP values (Fig 4A).

The analysis of “anti-TcI TRYPO reactivity at 1:4,000” revealed a clear polarization of serum samples from hosts infected with TcI/Colombiana strain with a unimodal distribution in the region of PPFP values around 100%, contrasting with a unimodal distribution in the region of PPFP values around 0% observed for serum samples from hosts infected with TcII/Y strain. Again, a typical bimodal distribution was noticed for serum samples from hosts infected with TcVI/CL strain (Fig 4B).

The histogram distribution of “anti-TcVI EPI reactivity at 1:1,000” revealed a clear Gaussian unimodal distribution of serum samples from hosts infected with TcI/Colombiana and TcVI/CL strains within the region of PPFP values around 50%. On the other hand, the unimodal distribution observed for serum samples from hosts infected with TcII/Y strain showed a clear shift towards PPFP values < 50% (Fig 4C).

Comparative analysis of median reactivity pattern of the selected pairs of attributes confirmed the trend observed by histogram and non-linear regression analysis, pointing out the ability of “anti-TcII AMA reactivity at 1:1,000” to segregate hosts infected with TcII/Y strain (and TcVI/CL strain) apart from those infected with TcI/Colombiana strain (Fig 4D). On the other hand, the “anti-TcI TRYPO reactivity at 1:4,000” was able to segregate hosts infected with TcI/Colombiana strain (and TcVI/CL strain) apart from those infected with TcII/Y strain (Fig 4E). Moreover, the “anti-TcVI EPI reactivity at 1:1,000” was capable to discriminate the hosts infected with TcVI/CL strain (and TcI/Colombiana strain) apart from those infected with TcII/Y strain (Fig 4F).

### Establishment of cut-off edges and performance of TcI/TcVI/TcII Chagas-Flow ATE-IgG2a for genotype-specific diagnosis of *T*. *cruzi* infection

Aiming at making the TcI/TcVI/TcII Chagas-Flow ATE-IgG2a applicable to the genotypic-specific diagnosis of *T*. *cruzi* infection, the overlaid trendlines for the overall reactivity (S1A Fig) along with the global median PPFP value of each pair of pre-selected attributes (S1B Fig) were employed to establish potential cut-off edges to categorize individual samples as they present negative (<cut-off) or positive (>cut-off) reactivity.

Using this approach, specific cut-off edges were defined for each pre-selected pairs of attributes (“anti-TcII AMA reactivity at 1:1,000”; “anti-TcI TRYPO reactivity at 1:4,000” and “anti-TcVI EPI reactivity at 1:1,000”), comprising PPFP = 40%, PPFP = 50% and PPFP = 45%, respectively (S1B Fig).

Diagrams were used to compile the reactivity patterns and calculate the proportion of negative and positive results for each selected set of attributes (“target-antigen/serum dilution/cut-off”). Data analysis showed that the set of attributes “anti-TcII AMA reactivity at 1:1,000, cut-off = 40%” were able to show positive results in 74% of hosts infected with TcII/Y strain (and 55% of TcVI/CL strain) apart from 14% of those infected with TcI/Colombiana strain (S1C Fig). Moreover, the set of attributes “anti-TcI TRYPO reactivity at 1:4,000, cut-off = 50%” showed positive results in 83% of hosts infected with TcI/Colombiana strain (and 59% of TcVI/CL strain) contrasting with 14% of those infected with TcII/Y strain (S1C Fig). Furthermore, the set of attributes “anti-TcVI EPI reactivity at 1:1,000, cut-off = 45%” showed positive results in 72% of hosts infected with TcI/Colombiana strain (and 55% of TcVI/CL strain) distinct from 27% of those infected with TcII/Y strain (S1C Fig).

Scatter plot distribution further illustrated the pre-selected sets of attributes segregated the reactivity of hosts infected with distinct *T*. *cruzi* genotypes, emphasizing the performance of “anti-TcII AMA reactivity at 1:1,000, cut-off = 40%” to discriminate the majority of the hosts infected with TcI/Colombiana strain and the ability of “anti-TcI TRYPO reactivity at 1:4,000, cut-off = 50%” and “anti-TcVI EPI reactivity at 1:1,000, cut-off = 45%” to discriminate the majority of the hosts infected with TcII/Y strain. In general, considerable proportion of hosts infected with the hybrid TcVI/CL strain presented positive results using the pre-selected set of attributes (S1D Fig).

### Performance of combined TcI/TcVI/TcII Chagas-Flow ATE-IgG2a for genotype-specific diagnosis of *T*. *cruzi* infection in two population/prototype scenarios

**Fig 5:**
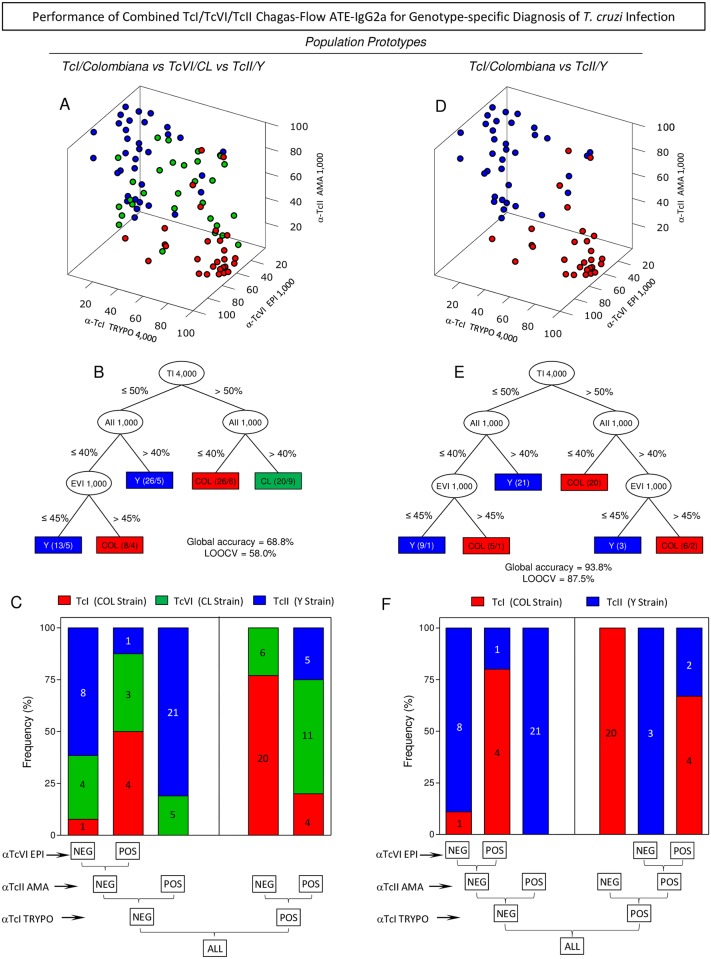
Performance of combined TcI/TcVI/TcII Chagas-Flow ATE-IgG2a for genotype-specific diagnosis of *T*. *cruzi* infection in two population/prototype scenarios. 3D plots were employed to identify clusters of TcI/TcVI/TcII Chagas-Flow ATE-IgG2a reactivity amongst sera samples from *T*. *cruzi*-infected mice in two population/prototypes including (A) TcI/Colombiana (red circle, n = 29) *vs* TcVI/CL (green circle, n = 29) *vs* TcII/Y (blue circle, n = 35) or (D) TcI/Colombiana (red circle, n = 29) *vs* TcII/Y(blue circle, n = 35), using the three selected pair of attributes (“target-antigen/serum dilution”). Data are expressed as Log of PPFP values for anti-TcI TRYPO at 1:4,000 (left lateral axis), anti-TcII AMA at 1:1,000 (vertical axis) and anti-TcVI EPI at 1:1,000 (right lateral axis). Decision trees were constructed using the set of attributes (“target-antigen/serum dilution/cut-off”) to create algorithms (root and branch attributes) to classify *T*. *cruzi* infected mice in a population/prototype including (B) TcI/Colombiana (red rectangle) *vs* TcVI/CL (green rectangle) *vs* TcII/Y (blue rectangle) or including (E) TcI/Colombiana (red rectangle) *vs* TcII/Y(blue rectangle). Global accuracy and leave-one-out-cross-validation-LOOCV are provided in the Figure. Bar charts representing the performance of the decision trees demonstrate the number of animals that ranked within each branch amongst the *T*. *cruzi*-infected hosts for (C) (TcI/Colombiana = red bar *vs* TcVI/CL = green bar *vs* TcII/Y = blue bar) and (F) (TcI/Colombiana = red bar *vs* TcII/Y = blue bar).

The performance of combined TcI/TcVI/TcII Chagas-Flow ATE-IgG2a was evaluated in two population/prototypes (TcI/Colombiana *vs* TcVI/CL *vs* TcII/Y strains and TcI/Colombiana *vs* TcII/Y strains) selected to exemplify the distribution of human *T*. *cruzi* infection in distinct geographical regions around the world. Data analysis was carried out using the sets of pre-selected attributes (“anti-TcII AMA reactivity at 1:1,000, cutoff = 40%”; “anti-TcI TRYPO reactivity at 1:4,000, cut-off = 50%” and “anti-TcVI EPI reactivity at 1:1,000, cut-off = 45%”), as presented in the Fig 5.

Three-dimensional plots were built to obtain a panoramic snapshot provided by the combined reactivity of the three sets of pre-selected attributes (Fig 5A and 5D). Data analysis was carried out in two population/prototypes (TcI/Colombiana *vs* TcVI/CL *vs* TcII/Y strains, Fig 5A) and (TcI/Colombiana *vs* TcII/Y strains, Fig 5D).

The results obtained for the first population/prototype (TcI/Colombiana *vs* TcVI/CL *vs* TcII/Y strains) demonstrated clearly that sera samples from hosts infected with TcI/Colombiana strain was confined in a region of high “anti-TcI TRYPO reactivity at 1:4,000” (left lateral axis) and low “anti-TcII AMA reactivity at 1:1,000” (vertical axis). In contrast, sera from hosts infected with TcII/Y strain presented a shift towards lower “anti-TcI TRYPO reactivity at 1:4,000” (left lateral axis) and higher “anti-TcII AMA reactivity at 1:1,000” (vertical axis). A slight translocation of samples from hosts infected with TcII/Y strain towards lower “anti-TcVI EPI reactivity at 1:1,000” (right lateral axis) was also observed. A notable evidence was that the sera samples from hosts infected with TcVI/CL strain displayed a typical interweaved distribution pattern (Fig 5A).

The dichotomic reactivity pattern of the three sets of pre-selected attributes was more evident when data analysis was performed in the second population/prototype which included only hosts infected with TcI/Colombiana *vs* TcII/Y strains (Fig 5D).

Decision tree analyses were built for the two population/prototypes (Fig 5B and 5E). The algorithm proposed for the first population/prototype indicated the “anti-TcI TRYPO reactivity at 1:4,000, cut-off = 50%” as the root attribute, followed by “anti-TcII AMA reactivity at 1:1,000, cut-off = 40%” as the first branch and “anti-TcVI EPI reactivity at 1:1,000, cut-off = 45%” as the second branch to classify sera samples from hosts infected with TcI/Colombiana *vs* TcVI/CL *vs* TcII/Y strains with a moderate global accuracy (68.8%, LOOCV = 58.0%) (Fig 5B). Data obtained for the second population/prototype indicated that the same decision tree algorithm presented high global accuracy (93.8%, LOOCV = 87.5%) (Fig 5E).

Bar charts were constructed to illustrate the categorical classification proposed by the decision trees, demonstrating the number of animals that ranked within each branch amongst the *T*. *cruzi*-infected hosts for the first population/prototype (TcI/Colombiana *vs* TcVI/CL *vs* TcII/Y strains, Fig 5C) and the second population/prototype (TcI/Colombiana *vs* TcII/Y strains, Fig 5F). Data demonstrated that the algorithm applied to the first population/prototype was not able to clusterize the serum samples from hosts infected with the TcVI/CL strain that display a spread ranking within branches (Fig 5C). On the other hand, the algorithm applied to the second population prototype yielded lower classification error with only four samples misplaced within branches (one sample from TcI/Colombiana strain and three from TcII/Y strain) (Fig 5F).

Discriminant analysis of combined TcI/TcVI/TcII Chagas-Flow ATE-IgG2a for genotype-specific diagnosis of *T*. *cruzi* infection groups performed for the two population/prototypes are provided in the S2 Fig. Data analysis demonstrate that for the first population/prototype, the combined TcI/TcVI/TcII Chagas-Flow ATE-IgG2a categorize 82.8% of serum samples from hosts infected with TcI/Colombiana strain apart from 82.9% of those infected with TcII/Y strain. However, only 38% of serum samples from hosts infected with TcVI/CL strain were clusterized in a particular branch (S2A Fig). If we consider the scenario represented by the second population/prototype, the data showed that 96.6% of serum samples from samples TcI/Colombiana strain were correctly classified apart from 91.4% of those infected with TcII/Y strain (S2B Fig).

### Criteria to define universal and genotype-specific diagnosis of *T*. *cruzi* infection by TcI/TcVI/TcII Chagas-Flow ATE-IgG2a in two population/prototype scenarios

**Fig 6:**
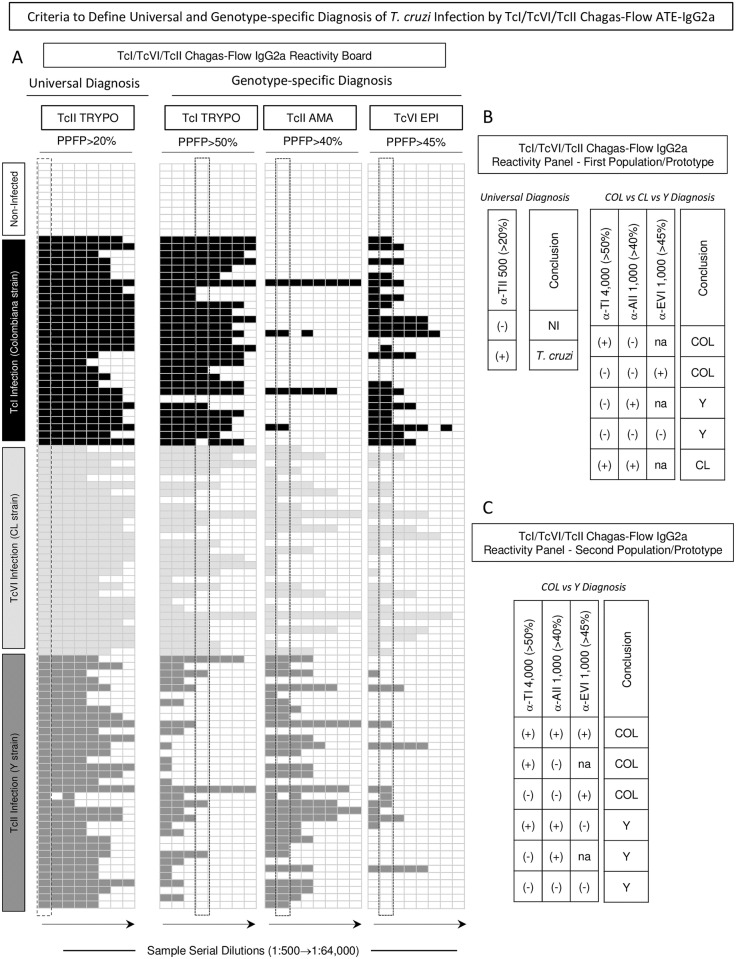
Criteria to define universal and genotype-specific diagnosis of *T*. *cruzi* infection by TcI/TcVI/TcII Chagas-Flow ATE-IgG2a in two population/prototype scenarios. (A) Reactivity boards were built to provide a panoramic snapshot of TcI/TcVI/TcII Chagas-Flow ATE-IgG2a applied to the universal and genotype-specific diagnosis of *T*. *cruzi* infection. Data mining approaches were used to pre-select the target-antigens and specific cut-off edges to define positive results for universal diagnosis purpose (TcII TRYPO, PPFP>20%) and genotype-specific diagnosis criteria (TcI TRYPO, PPFP>50%; TcII AMA, PPFP>40% and TcVI EPI, PPFP>45%) based on the differential positive reactivity of sera samples (TcI/Colombiana strain = black rectangle, TcVI/CL strain = light gray rectangle and TcII/Y strain = dark gray rectangle) from negative reactivity (white rectangle) observed for *T*. *cruzi* infected hosts and non-infected mice. The pre-selected sera dilutions defined by decision tree analysis are underscored by dotted rectangles and include TcII TRYPO PPFP>20% at 1:500 for universal diagnosis and the set of attributes (TcI-TRYPO/PPFP>50%/4,000 followed by TcII-AMA/PPFP>40%/1,000 and TcVI-EPI/PPFP>45%/1,000) for genotype-specific diagnosis criteria. Reactivity panels were constructed to define the diagnosis conclusion when applying TcI/TcVI/TcII Chagas-Flow ATE-IgG2a for (B) universal diagnosis of *T*. *cruzi* infection and genotype-specific diagnosis in a population/prototype including TcI/Colombiana strain (COL), TcVI/CL strain (CL) or TcII/Y (Y) strain or (C) including TcI/Colombiana strain (COL) or TcII/Y strain (Y).

Reactivity boards were constructed using the pre-selected set attributes, including “anti-TcII TRYPO reactivity at 1:500, cut-off = 20%” for universal diagnosis purpose and “anti-TcI TRYPO reactivity at 1:4,000, cut-off = 50%”, “anti-TcII AMA reactivity at 1:1,000, cut-off = 40%” and “anti-TcVI EPI reactivity at 1:1,000, cut-off = 45%” for genotype-specific diagnosis (Fig 6). The reactivity at selected serum dilutions (Fig 6—dashed frames) were used to further create a receiver operating reactivity panel, applicable for universal diagnosis (NI vs *T*. *cruzi*-infected hosts, Fig 6B) and for genotype-specific diagnosis applied to the first population/prototype (TcI/Colombiana *vs* TcVI/CL *vs* TcII/Y strains, Fig 6B) and the second population/prototype (TcI/Colombiana *vs* TcII/Y strains, Fig 6C).

When using the TcI/TcVI/TcII Chagas-Flow ATE-IgG2a applied for the universal diagnosis purpose, the attribute α-TII 500 (>20%) presenting a positive score (+) define the presence of *T*. *cruzi* infection, while a negative score (-) ruled out the presence of *T*. *cruzi* infection.

The reactivity panel for TcI/TcVI/TcII Chagas-Flow ATE-IgG2a applied for the genotype-specific diagnosis regardless the population/prototype scenario indicated scores sequences of α-TI (trypomastigote TcI) 4,000(>50%)/α-AII (amastigote TcII) 1,000(>40%)/α-EVI (epimastigote TcVI) 1,000(>45%) to define the *T*. *cruzi* infection with distinct genotypes defined as: (+/-/not applicable (na) or -/-/+) for TcI/Colombiana and (-/+/na or -/-/-) for TcII/Y strain infection. The extensions of the score (+/+) do not allow the proper identification the *T*. *cruzi* infection genotype, since in can belongs to hosts infected with TcI/Colombiana (+/+/+), TcVI/CL (+/+/na) or even TcII/Y strain (+/+/-) (Fig 6B and 6C).

Together, the proposed receiver operating reactivity panels for TcI/TcVI/TcII Chagas-Flow ATE-IgG2a provided a feasible tool to classify the serum samples as they belong to the true respective groups, supporting the potential of this method for universal and genotype-specific diagnosis of *T*. *cruzi* infection.

## Discussion

The broad genetic variability of *T*. *cruzi* has been related to biological characteristics (infectivity, parasitemia, tissue tropism, mortality during the acute phase of infection [13,26–30] and susceptibility/resistance to drugs [5,8,31–34] in murine model and infectivity, replication and differentiation in vector o) [35], epidemiological characteristics [4,36,37] and clinical manifestations [38–40] of Chagas disease. Therefore, the knowledge of parasite genetics may offer insights about the biology of the parasite, patient’s treatment outcome, clinical aspects of human disease, as well as how to establish epidemiological surveillance and control of Chagas disease [41, 42]. So, the genetic diversity of *T*. *cruzi* infection may also influence the sensitivity of the techniques used for Chagas disease diagnosis [43–45].

The currently available methods for genotype-specific diagnosis of *T*. *cruzi* infection, most based on molecular biology approaches present distinct levels of complexity and in general display high specificity but moderate sensitivity [4, 10–12, 46–52]. Moreover, a combination of several genetic markers is necessary to detect and distinguish the *T*. *cruzi* genotypes [10–12]. Furthermore, the majority of these methods can not directly be performed in biological and clinical samples, requiring previous parasite isolation by hemoculture/xenoculture followed by *in vitro* growth and maintenance that may lead to clonal selection [14,53–55]. Besides, it is known that the parasitemia in patients and reservoirs of *T*. *cruzi* are variable and that the success of parasite isolation is dependent on the host parasitemia. The full extent of lineage distribution in nature using genetic markers it is not known due to the low levels of circulating parasitemia and possible lineage-specific tissue sequestration [34,56–58]. In addition, it has been proposed that parasite isolates from blood may not necessarily represent the full set of strains current in the individual, hence some strains of *T*. *cruzi* can be confined to tissues [11,13,16,15]. In general, PCR (polymerase chain reaction) based genotyping has limitations that hamper the analysis of large numbers of samples. Therefore, the development of methods for diagnosis and serotyping of Chagas disease are urgently required.

Attempting to address this matter, Mendes *et al*. (2013) [18] have described a set of B-cell epitopes able to discriminate TcI and TcII infections, demonstrating the potential of these targets for Chagas disease serotyping. Later on, the putative TcI epitope reported by Mendes *et al*. (2013) [18] was found to be conserved across all *T*. *cruzi* lineages by studies developed by Bhattacharyya *et al*. (2014) [19]. Moreover, samples from animals infected with TcVI presented cross-reactivity with a range of *T*. *cruzi*-derived peptides, suggesting the need of improved antigen search and the development of a robust panel of strain-specific epitopes to achieve a method applicable in large epidemiological studies [18].

Aiming at developing innovative serological approaches for universal and improved genotypic-specific diagnosis of *T*. *cruzi* experimental infection, our goal was optimize the Chagas-Flow ATE methodology, proposed originally by Alessio *et al*. (2014) [21]. The present approach is based on parallel batches of distinct *T*. *cruzi* genotypes as target antigens, employing parasites strains of three more important genotypes associated with human infection and Chagas disease (TcI, TcVI and TcII) [4,38,59] although others genotypes exist such as TcIII [60–62], TcIV recently described as associated to oral transmission [63, 64] and TcV associated to the classical clinical forms of Chagas disease (cardiac and digestive) [39].

Previous studies have demonstrated that patients from distinct geographic areas, infected with different genotypes of *T*. *cruzi* seem to display differential serological pattern upon serodiagnosis of Chagas disease, when employing distinct methodological approaches and different *T*. *cruzi* target antigens [4,43,44,65]. Verani *et al*. (2009) [44] have demonstrated that the performance of two serological tests, using serum samples from distinct geographical regions (Bolivia and Peru) displayed distinct sensitivity, ranging from 26.6%-87.5%, corroborating the hypothesis that intrinsic features of regional parasite strains may influence the serological tests. Studies using six recombinant antigens of *T*. *cruzi* tested in samples from Argentina, Brazil, Chile, Colombia, El Salvador, Guatemala, Honduras and Venezuela also reported discrepancy in the serological reactivity ranging from 79% to 100% [43].

In this study, we have intended to evaluate the performance of combined TcI/TcVI/TcII Chagas-Flow ATE-IgG2a for universal diagnosis of *T*. *cruzi* infection, simulating two population prototypes that may represent the geographic distribution of *T*. *cruzi* infection in the Latin America. The first population prototype, represent regions were TcI, TcVI and TcII genotypes are co-endemic. In the second population setting, we intended to evaluate the performance of combined TcI/TcVI/TcII Chagas-Flow ATE-IgG2a to discriminate the infections with TcI/Colombiana *vs* TcII/Y strains. Our findings demonstrated that regardless the population prototype, the TcI/TcVI/TcII Chagas-Flow ATE-IgG2a presented an outstanding performance for universal diagnosis of *T*. *cruzi* infection using the set of attributes “anti-TcII TRYPO reactivity at 1:500, cut-off = 20%” (Fig 3). In fact, although the sensitivity of TcI/TcVI/TcII Chagas-Flow ATE-IgG2a varies according to the target antigen employed, the TcII TRYPO antigen was able to detect seroreactivity in all mice infected with distinct *T*. *cruzi* genotypes (Figs 2 and 3). Corroborating with our study, Bhattacharyya *et al*. (2014) [19] also observed that all sera from patients with chronic Chagas disease recognized the *T*. *cruzi* TcII lysate antigen preparation.

Previous studies have also demonstrated the influence of parasite genotype on the pattern of antibody response in experimental models [66]. It has been described that the profile of lytic antibodies varies when distinct *T*. *cruzi* strains are used as targets, suggesting that genotypic-specific antigenic features may be involved in the induction of lytic antibodies [67–69]. Moreover, Di Noia *et al*. (2002) [70] have reported that two *T*. *cruzi* antigens, named small surface antigen of trypomastigotes (TSSAI and TSSAII) presented the ability of genotypic-specific recognition of *T*. *cruzi* infection. These studies were expanded by Bhattacharyya *et al*. (2010, 2014 and 2015) [17,19,20] that reported that TSSA pepII/V/VI isoforms were able to distinguish samples of hosts infected with distinct *T*. *cruzi* genotypes. Based on these findings, the authors proposed that TSSA isoforms are feasible serological markers to identify a *T*. *cruzi* lineage in human and experimental infection. However, the TSSA pepI did not yield significant reactivity, suggesting that novel targets for TcI is still required.

The innovative TcI/TcVI/TcII Chagas-Flow ATE-IgG2a methodology presented a high-quality performance to segregate infections with TcI/Colombiana, TcVI/CL or TcII/Y strain. The performance of combined TcI/TcVI/TcII Chagas-Flow ATE-IgG2a for genotype-specific diagnosis of *T*. *cruzi* infection differs depending on the population prototypes used to represent distinct geographic regions of *T*. *cruzi* infection in the Latin America. In the first prototype (TcI/TcVI/TcII), our data demonstrated that the proposed method showed a moderate global accuracy (68.8%, LOOCV = 58.0%) to discriminate the infections with TcI/Colombiana *vs* TcVI/CL *vs* TcII/Y strains. On the other hand, the combined TcI/TcVI/TcII Chagas-Flow ATE-IgG2 was capable to discriminate the infections with TcI/Colombiana *vs* TcII/Y strains in the second population prototype (TcI/TcII) with high global accuracy (93.8%, LOOCV = 87.5%) (Fig 5). Overall, hosts infected with TcI/Colombiana and TcII/Y strains displayed opposite patterns of reactivity with “anti-TcI TRYPO” and “anti-TcII AMA” and hosts infected with TcVI/CL strain showed a typical interweaved distribution pattern (Figs 4 and S1). This phenomenon may reflect the phylogenetic origin of DTUs [4] where TcI and TcII are ancestral DTUs presenting polar characteristics [5,27,28,30,71–73], whereas the TcVI has a hybrid origin, showing intermediate characteristics of both polar genotypes [73,74].

In conclusion, based on the receiver operating characteristic, the TcI/TcVI/TcII Chagas-Flow ATE-IgG2a seems to be a feasible tool to classify the serum samples as they belong to the true respective groups infected with distinct *T*. *cruzi* genotypes (Fig 6), suggesting its applicability for both, universal and genotype-specific diagnosis of *T*. *cruzi* infection in clinical laboratories. The proposed methodology includes essential advantages such as high sensitivity and specificity, ease to perform, using a wide range of antigenic preparation into a single flow cytometric platform [21,75–79]. Future derivation of TcI/TcVI/TcII Chagas-Flow ATE-IgG2a as the development of a suitable ELISA or multiplex beads assay would contribute to practical applications in routine clinical laboratories, since the original version of this fluorescence-based methodology is more reliable for applications in reference laboratories. Additional tests are under investigation to establish accuracy of TcI/TcVI/TcII Chagas-Flow ATE-IgG2a to identify mixed infections with distinct *T*. *cruzi* genotypes. An extension of this study may be applicable to other genetic groups not included in this work (TcIII, TcIV and TcV). Further studies including serum samples from patients with genotypic-specific diagnosis Chagas disease performed by molecular methods are currently under investigation as a proof-of-concept to propose a prototype for clinical purposes, epidemiological studies and post-therapeutic monitoring applications.

## Supporting information

S1 Fig Cut-off edges and performance of TcI/TcVI/TcII Chagas-Flow ATE-IgG2a for genotype-specific diagnosis of *T*. *cruzi* infection. (A) The trendlines of anti-TcII AMA at 1:1,000, anti-TcI TRYPO at 1:4,000 and anti-TcVI EPI at 1:1,000 reactivity observed for *T*. *cruzi*-infected hosts, (TcI/Colombiana strain = black dashed line, TcVI/CL strain = light gray dashed line and TcII/Y strain = dark gray dashed line) were overlaid aiming to differentiate the reactivity pattern. The results were expressed as the proportion of samples displaying a given PPFP values amongst *T*. *cruzi*-infected hosts. (B) The whole set of reactivity data were used to calculate the global median PPFP values applied as the cut-off edge to segregate the individual samples as they present negative (white square) or positive (black square) reactivity at the selected target-antigen/serum dilution. The results were expressed as the range of PPFP values in scatter plots for individual serum samples (C) Diagrams were used to compile the reactivity patterns and calculate the proportion of negative and positive results for each selected set of attributes (“target-antigen/serum dilution/cut-off”). (D) Representative scatter plots were also used to illustrate the ability of each set of attributes (“target-antigen/serum dilution/cut-off”) to discriminate the reactivity of serum samples amongst the *T*. *cruzi*-infected mice (TcI-infection/Colombiana, n = 29; TcVI/CL, n = 29 and TcII/Y, n = 35). The results were expressed as the range of PPFP values in scatter plots for individual serum samples. The dotted line represents the cut-off for each target-antigen/serum dilution. Clusters of distinct reactivity patterns are highlighted by light-gray doted frames whereas indiscriminate distribution pattern underscore by white-background frame.
(TIF)

S2 Fig Discriminant analysis of combined TcI/TcVI/TcII Chagas-Flow ATE-IgG2a for genotype-specific diagnosis of *T*. *cruzi* infection. (A) Discriminant analyses of combined TcI/TcVI/TcII Chagas-Flow ATE-IgG2a were performed for genotype-specific diagnosis of *T*. *cruzi* infection in a population/prototype including TcI/Colombiana strain, TcVI/CL strain or TcII/Y strain. (B) Discriminant analyses of combined TcI/TcVI/TcII Chagas-Flow ATE-IgG2a were performed for genotype-specific diagnosis of *T*. *cruzi* infection in a population/prototype including TcI/Colombiana strain or TcII/Y strain. The global accuracy and leave-one-out-cross-validation-LOOCV provided in the Figure.
(TIF)
