# Supplementary figures and images for: Performance of TcI/TcVI/TcII Chagas-Flow ATE-IgG2a for universal and genotype-specific serodiagnosis of Trypanosoma cruzi infection
Source: PLoS Negl Trop Dis. 2017 Mar 23;11(3):e0005444. doi: 10.1371/journal.pntd.0005444 (PMC5380352; doi:10.1371/journal.pntd.0005444)

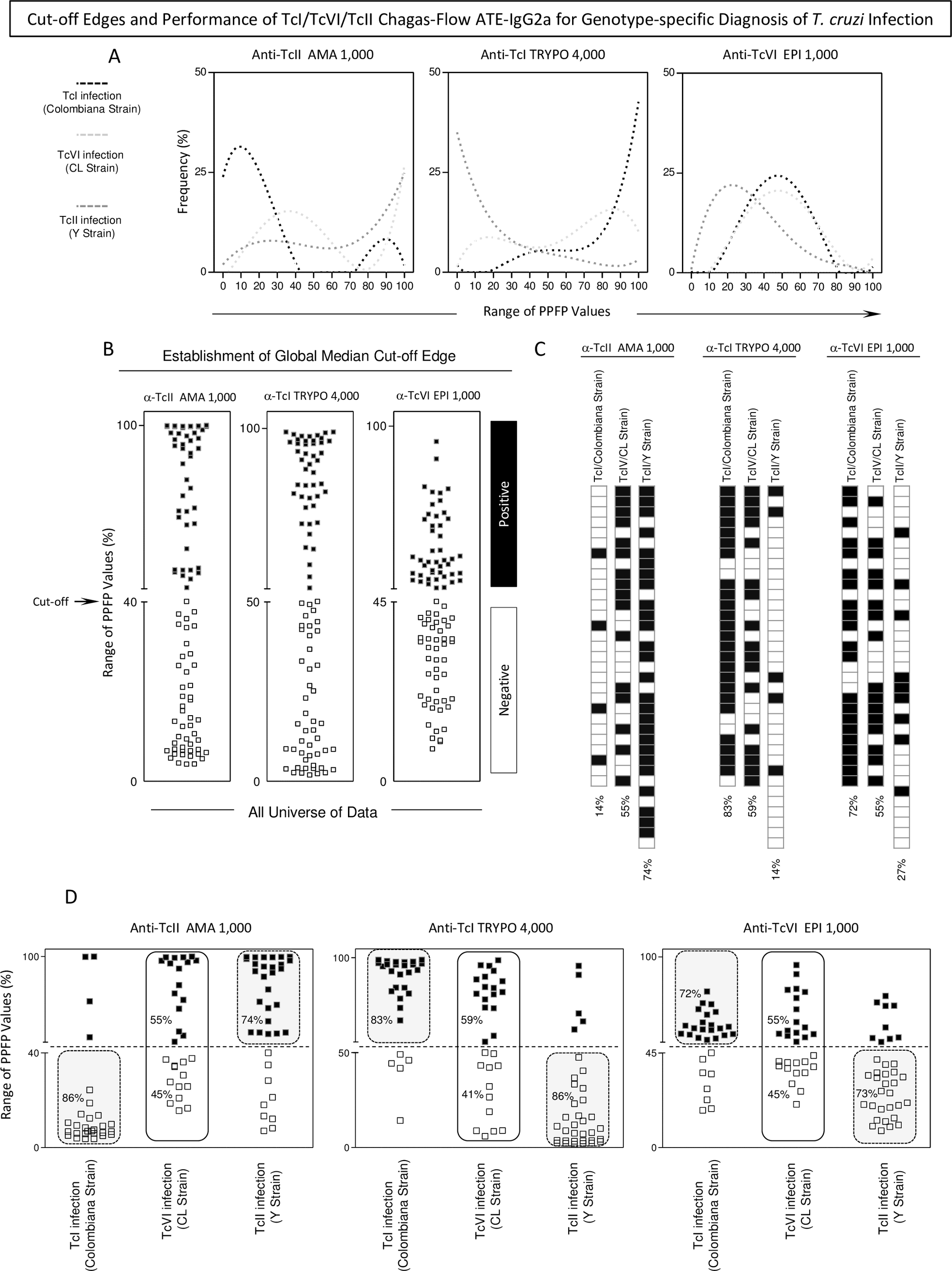

Supplement: S1 Fig — (A) The trendlines of anti-TcII AMA at 1:1,000, anti-TcI TRYPO at 1:4,000 and anti-TcVI EPI at 1:1,000 reactivity observed for T. cruzi-infected hosts, (TcI/Colombiana strain = black dashed line, TcVI/CL strain = light gray dashed line and TcII/Y strain = dark gray dashed line) were overlaid aiming to differentiate the reactivity pattern. The results were expressed as the proportion of samples displaying a given PPFP values amongst T. cruzi-infected hosts. (B) The whole set of reactivity data were used to calculate the global median PPFP values applied as the cut-off edge to segregate the individual samples as they present negative (white square) or positive (black square) reactivity at the selected target-antigen/serum dilution. The results were expressed as the range of PPFP values in scatter plots for individual serum samples (C) Diagrams were used to compile the reactivity patterns and calculate the proportion of negative and positive results for each selected set of attributes (“target-antigen/serum dilution/cut-off”). (D) Representative scatter plots were also used to illustrate the ability of each set of attributes (“target-antigen/serum dilution/cut-off”) to discriminate the reactivity of serum samples amongst the T. cruzi-infected mice (TcI-infection/Colombiana, n = 29; TcVI/CL, n = 29 and TcII/Y, n = 35). The results were expressed as the range of PPFP values in scatter plots for individual serum samples. The dotted line represents the cut-off for each target-antigen/serum dilution. Clusters of distinct reactivity patterns are highlighted by light-gray doted frames whereas indiscriminate distribution pattern underscore by white-background frame. (TIF) [file pntd.0005444.s001.tif]

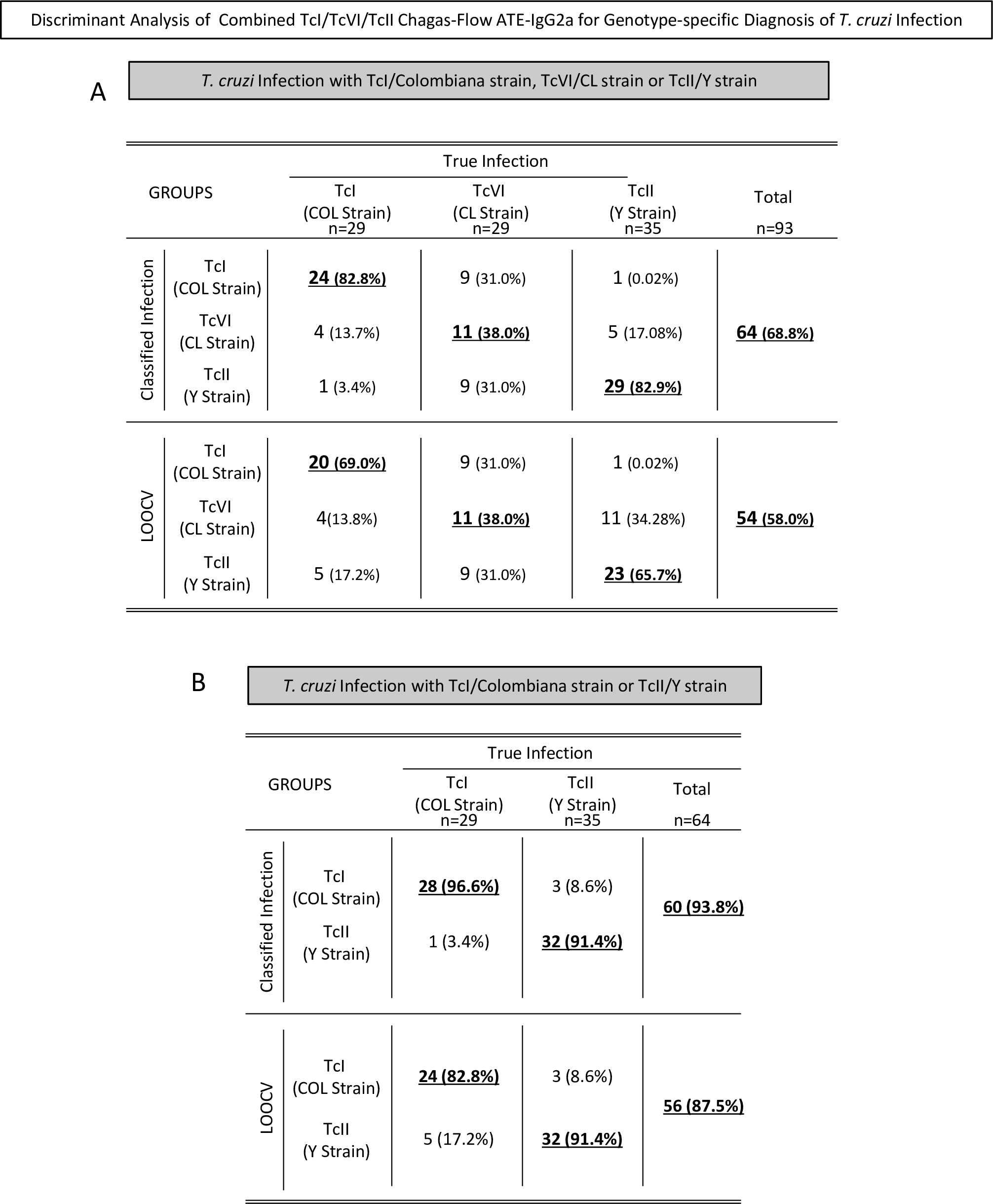

Supplement: S2 Fig — (A) Discriminant analyses of combined TcI/TcVI/TcII Chagas-Flow ATE-IgG2a were performed for genotype-specific diagnosis of T. cruzi infection in a population/prototype including TcI/Colombiana strain, TcVI/CL strain or TcII/Y strain. (B) Discriminant analyses of combined TcI/TcVI/TcII Chagas-Flow ATE-IgG2a were performed for genotype-specific diagnosis of T. cruzi infection in a population/prototype including TcI/Colombiana strain or TcII/Y strain. The global accuracy and leave-one-out-cross-validation-LOOCV provided in the Figure. (TIF) [file pntd.0005444.s002.tif]
